# Critical behavior of the quasi-two-dimensional semiconducting ferromagnet CrSiTe_3_

**DOI:** 10.1038/srep33873

**Published:** 2016-09-21

**Authors:** Bingjie Liu, Youming Zou, Lei Zhang, Shiming Zhou, Zhe Wang, Weike Wang, Zhe Qu, Yuheng Zhang

**Affiliations:** 1 High Magnetic Field Laboratory, Chinese Academy of Sciences, Hefei, Anhui 230031, China; 2 University of Science and Technology of China, Hefei, Anhui 230026, China; 3 Hefei National Laboratory for Physical Sciences at Microscale, University of Science and Technology of China, Hefei, Anhui 230026, China

## Abstract

The semiconducting ferromagnet CrSiTe_3_ is a promising candidate for two-dimensional magnet simply by exfoliating down to single layers. To understand the magnetic behavior in thin-film samples and the possible applications, it is necessary to establish the nature of the magnetism in the bulk. In this work, the critical behavior at the paramagnetic to ferromagnetic phase transition in single-crystalline CrSiTe_3_ is investigated by bulk magnetization measurements. We have obtained the critical exponents (*β* = 0.170 ± 0.008, *γ* = 1.532 ± 0.001, and *δ* = 9.917 ± 0.008) and the critical temperature *T*_*C*_ = 31.0 K using various techniques such as modified Arrott plot, Kouvel-Fisher plot, and critical isotherm analysis. Our analysis suggests that the determined exponents match well with those calculated from the results of renormalization group approach for a two-dimensional Ising model coupled with long-range interaction.

Two-dimensional (2D) materials have attracted significant attention because of the emergence of novel physics and potential applications^1^,^2^,^3^,^4^,^5^. One of the primary goals in this area is to develop ferromagnetic (FM) semiconductors, which not only are eagerly needed in next-generation nano-spintronics^6^,^7^,^8^, but also exhibit unusual magnetism that are of great interest on its own^9^. Within this context, the intrinsic semiconducting ferromagnet CrSiTe_3_ has generated considerable interest recently because first principle calculations predict the important coexistence of ferromagnetic and semiconducting properties upon exfoliating down to single layers in this material^10^. More interestingly, the Curie temperature in single layers is predicted to be higher than that in bulk, and to further increase when CrSiTe_3_ single layers are strained^10^,^11^,^12^.

To understand the magnetic behavior in thin-film samples and the possible applications of this material, it is necessary to establish the nature of the magnetism in the bulk. Previous studies find that it undergoes a paramagnetic (PM) to FM phase transition around 33 K and shows a strong coupling between magnetic and lattice degrees of freedom^13^. Nevertheless, the nature of the PM-FM phase transition is not fully understood yet. Early neutron measurements found a critical exponent *β* ≈ 0.17 and a spin gap of ~6 meV^14^. Based on these results, they suggested CrSiTe_3_ to be a rare example of the quasi-2D Ising ferromagnet^14^. Recent neutron work observed a critical exponent *β* ≈ 0.151 (2) close to the value expected for a 2D phase transition^15^. However, based on the spin wave analysis, they argued that the spins should be Heisenberg-like^15^. These controversial results prompt us to perform an extensive magnetization measurement to investigate the critical behavior of CrSiTe_3_, expecting the universality class to which the material belongs to gives important clues for the understanding of the unusual magnetism in this material. By performing critical analysis with various techniques, we have determined the critical exponents and the critical temperature for CrSiTe_3_. Our analysis indicate that the obtained critical exponents are in good agreement with those calculated from the results of renormalization group approach for 2D Ising model coupled with long-range interaction.

## Results and Discussion

According to the scaling hypothesis, the critical behavior of a magnetic system exhibiting a second-order magnetic phase transition near the Curie point can be characterized by a series of critical exponents^16^. The existence of a diverging correlation length 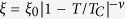 leads to universal scaling laws for the spontaneous magnetization *M*_*S*_(*T*) and the initial susceptibility *χ*_*0*_(*T*). The spontaneous magnetization *M*_*S*_(*T*) below *T*_*C*_, the inverse initial susceptibility *χ*_*0*_^−1^(*T*) above *T*_*C*_ and the measured magnetization *M*(*H*) at *T*_*C*_ are characterized by a set of critical exponents *β*, *γ*, and *δ*, respectively. The mathematical definitions of these exponents from magnetization are described as^17^:


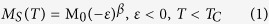



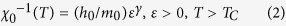



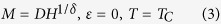


where *ε* = (T − T_*C*_)/T_*C*_ is the reduced temperature, and *M*_0_, *h*_0_/*m*_0_ and *D* are the critical amplitudes. Using scaling hypothesis, the relationship among the variables *M*(*H*, *ε*), *H* and *T* can be expressed as:


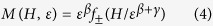


where *f*_+_ for *T* > *T*_*C*_ and *f*_−_ for *T* < *T*_*C*,_ respectively, are the regular functions. Furthermore, the renormalized magnetization 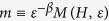 and the renormalized field 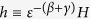 should follow two universal rules: one for *T* < *T*_*C*_ and the other for *T* > *T*_*C*_.

**Figure 1:**
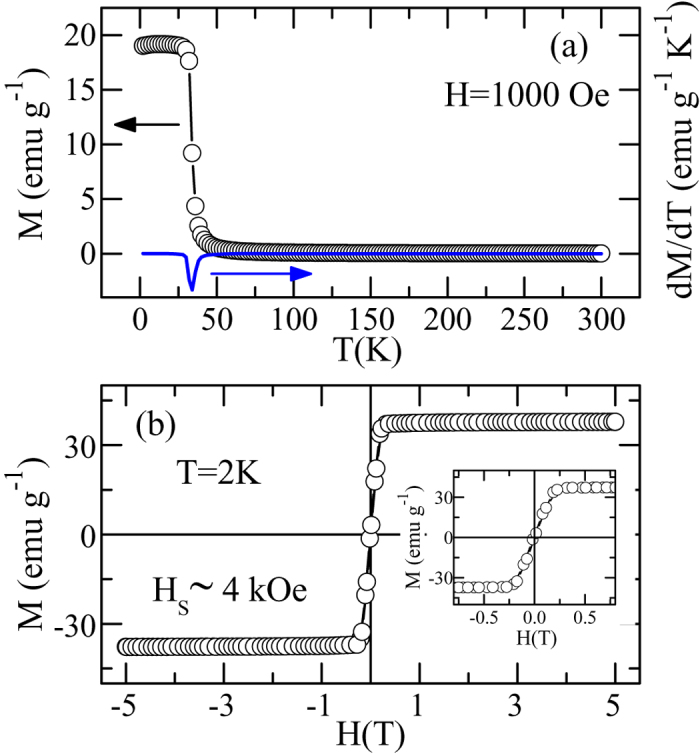
(**a**) The temperature dependence of magnetization *M*(*T*); (**b**) the isothermal magnetization *M*(*H*) at 2 K for CrSiTe_3_. The inset shows the enlarged view in the low field region.

Figure 1(a) shows the temperature dependence of magnetization *M*(*T*) under an applied field of 1000 Oe after the zero-field-cooling sequence (left coordinate). An abrupt PM-FM transition is observed to occur around 34 K. Curie-Weiss fitting to the magnetization above 150 K yields the Curie-Weiss temperature *θ* = 52(6) K. This is almost twice the value of *T*_C_, suggesting strong FM interactions in CrSiTe_3_. The effective moment is determined to be *μ*_*eff*_ = 4.0(4) *μ*_*B*_, which is close to the theoretical value expected for Cr^3+^ of 3.87 *μ*_*B*_. Figure 1(b) displays the isothermal magnetization *M*(*H*) at 2 K, which shows a typical FM behavior with the saturation field *H*_S_ ~4000 Oe. The inset to Fig. 1(b) shows the enlarged view of the *M*(*H*) at low fields. Little magnetic hysteresis is observed, which means almost zero coercive force in CrSiTe_3_. All these results are in good agreement with previous reports^13^.

**Figure 2:**
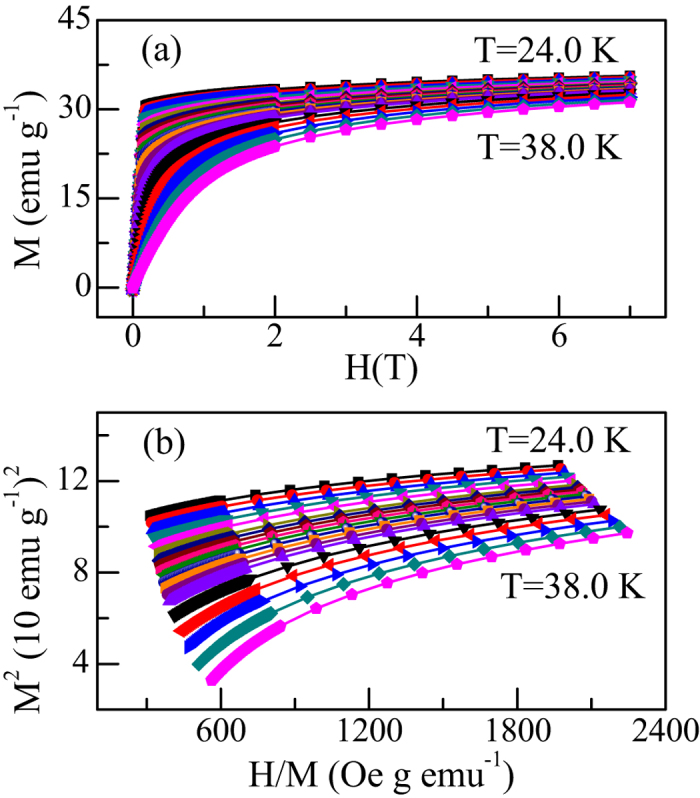
(**a**)Typical initial isotherm curves around *T*_*C*_ for CrSiTe_3_; (**b**) the Arrott plot (*M*^2^ vs. *H*/*M*) of isotherms around *T*_*C*_ for CrSiTe_3_.

Typical initial isotherm curves are shown in Fig. 2(a). Generally, one can obtain the critical exponents and the critical temperature by the Arrott plot analysis^18^. The Arrott plot assumes that the critical exponents follow the mean-field theory with the critical exponents *β* = 0.5 and *γ* = 1.0. Following this method, the *M*^2^ vs. *H*/*M* will show a set of parallel straight lines, and the isotherm at the critical temperature *T*_*C*_ should pass through the origin. Meanwhile, it can directly give *χ*_*0*_^−1^(*T*) and *M*_*S*_(*T*) as the intercept on *H*/*M* axis and on positive *M*^*2*^ axis, respectively. Moreover, according to the Banerjee’s criterion^19^, one can judge the order of the magnetic transition through the slope of the straight line: the positive slope corresponding to a second-order transition and the negative slope to a first-order one. Figure 2(b) shows the Arrott plot of CrSiTe_3_. Obviously, the positive slope in the Arrott plot clearly indicates that the PM-FM phase transition is a second-order one. However, all curves show nonlinear behavior, indicating that the mean-field model is not valid for CrSiTe_3_.

We also examined other three-dimensional (3D) models, including 3D-Heisenberg, 3D-XY, 3D-Ising and tricritical mean-field models^20^,^21^. As shown in Figure S1 in Supplementary Information, all these models failed to yield parallel straight lines, suggesting the breakdown of these 3D models.

**Figure 3:**
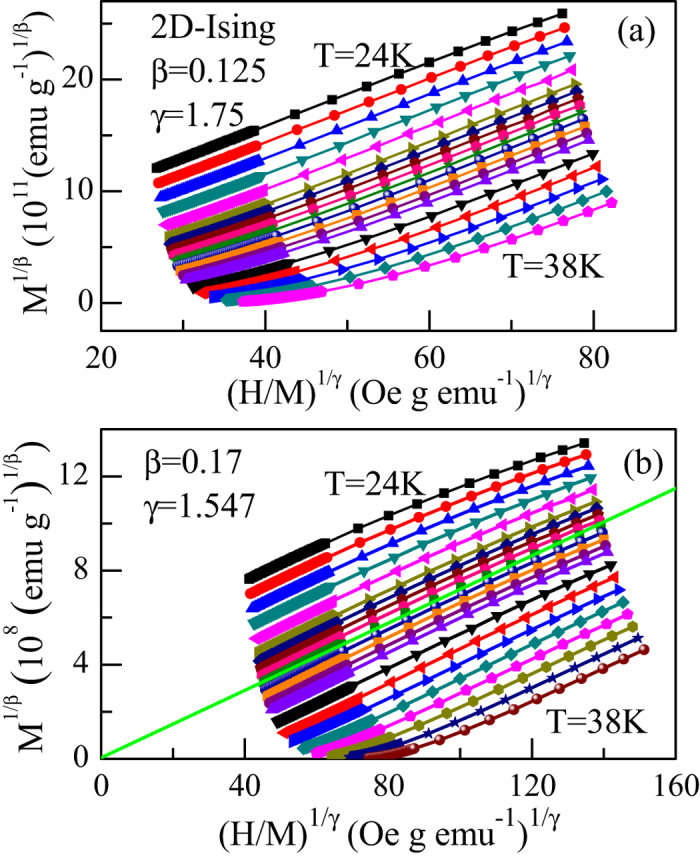
(**a**) The 2D-Ising model plot of isotherms for CrSiTe_3_; (**b**) the modified Arrott plot (*M*^1/*β*^ vs. (*H*/*M*)^1/*γ*^) of isotherms with *β* = 0.17 and *γ* = 1.547 for CrSiTe_3_. The straight line is the linear fit of isotherm at 31.0 K which almost passes through origin.

The failure of these 3D models might not be surprising, since CrSiTe_3_ was found to show strong 2D characteristics^14^,^15^. Hence, we further analyze the isothermal data with 2D-Ising model^22^, which are shown in Fig. 3(a). It can be clearly seen that there is a set of relatively parallel straight lines, indicating that the 2D-Ising model is much superior to those 3D models. However, it is noted that one still cannot find a single straight line that passes through origin, hinting that CrSiTe_3_ could not be rigorously described by the 2D-Ising model.

**Figure 4:**
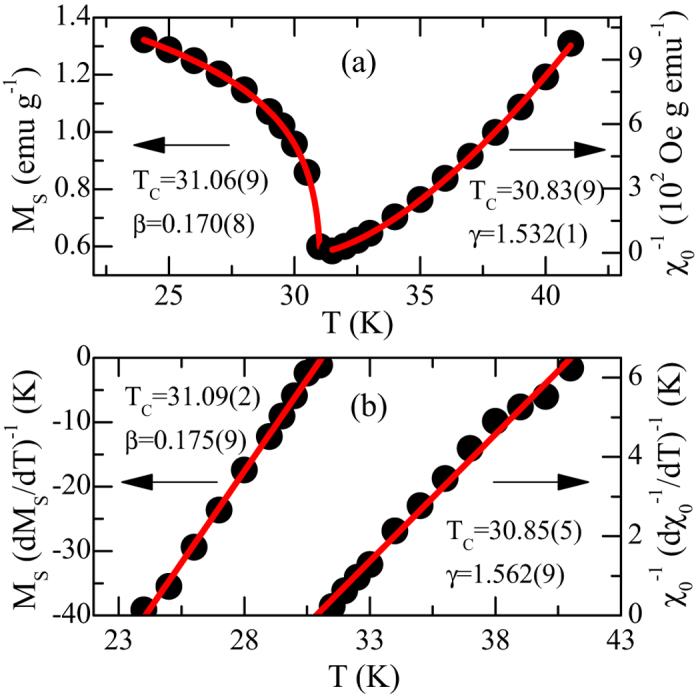
(**a**) The temperature dependence of *M*_S_ and *χ*_0_^−1^ for CrSiTe_3_ with the fitting solid lines; (**b**) the Kouvel-Fisher plot of spontaneous magnetization *M*_*S*_(*T*) (left axis) and inverse initial susceptibility *χ*_0_^−1^(*T*) (right axis) for CrSiTe_3_.

To clarify the critical behavior of CrSiTe_3_, we have taken recourse to which is commonly known as modified Arrott plot^23^. The modified Arrott plot is given by the Arrott-Noaks equation of state:


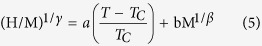


where *a* and *b* are considered to be constants. To find out the proper values of *β* and *γ*, a rigorous iterative method has been used^24^. The starting values of *M*_S_(*T*) and 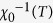 were determined from the 2D-Ising model plot (see Fig. 3(a)) following the Eqs (1) and (2). The obtained new values of *β* and *γ* were then used to figure out new modified Arrott plot. It should be mentioned that during fitting the straight lines, the critical temperature *T*_*C*_ is a free parameter and varied in order to get the best fitting results. This process was repeated until the iterations converge. After doing this exercise, the stable values of the critical exponents and the critical temperature have been obtained. Figure 3(b) displays the modified Arrott plot generated by using *β* = 0.17 and *γ* = 1.547. It is noted that at very low fields, the plotted isotherms are slightly curved as they represent averaging over domains magnetized in different directions^25^. Nevertheless, there is a set of reasonably good parallel straight lines. Moreover, the isotherm is found to pass through the origin at 31.0 K, which is the *T*_*C*_ of CrSiTe_3_. The finally obtained 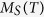 and 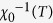 are plotted as a function of temperature in Fig. 4(a). Using these values of *M*_*S*_(*T*) and *χ*_*0*_^−1^(*T*), Eq. (1) gives *β* = 0.170(8), *T*_*C*_ = 31.06(9) K for *T* < *T*_*C*_ and Eq. (2) gives *γ* = 1.532(1), *T*_*C*_ = 30.83(9) K for *T* > *T*_*C*_, respectively. These estimated critical exponents and *T*_*C*_ from Eqs (1) and (2) are reasonably close to the values obtained from modified Arrott plot in Fig. 3(b).

To obtain more accurate values of the critical exponents as well as the critical temperature, we used the Kouvel-Fisher technique^26^. According to this method, *M*_S_(d*M*_S_/d*T*)^−1^ and *χ*_0_^−1^(d*χ*_0_^−1^/d*T*)^−1^ plotted against temperature should be straight lines with slopes 1/*β* and 1/*γ*, respectively. As shown in Fig. 4 (b), the linear fits to the data give *β* = 0.175(9), *T*_*C*_ = 31.09(2) K for *T* < *T*_*C*_ and *γ* = 1.562(9), *T*_*C*_ = 30.85(5) K for *T* > *T*_*C*_, respectively. It can be mentioned that values of the critical exponents as well as the critical temperature are not sensitive to the temperature range chosen (see Figure S3 and Table SI in Supplementary Information), indicating that they are reliable and unambiguous.

**Figure 5:**
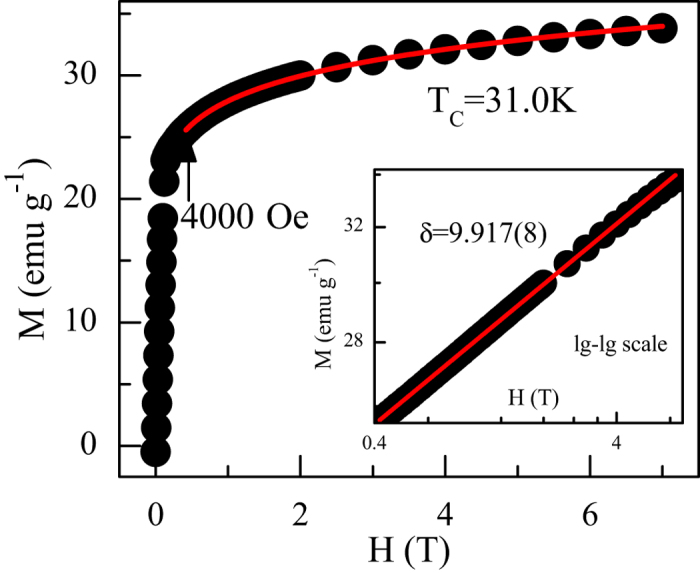
*M* vs. *H* plot collected at *T*_*C*_ (=31.0 K) for CrSiTe_3_. Inset shows the same plot in log-log scale and the straight line is the linear fit following Eq. (3). The critical exponent mentioned in graph is obtained from fitting of the data.

The third exponent *δ* can be determined from the critical isotherm analysis and the Widom scaling relation. Figure 5 shows the isotherm at *T*_*C*_ = 31.0 K and its inset shows the same plot in log-log scale. According to Eq. (3), the *M*(*H*) at the critical temperature should be a straight line in log-log scale with the slope 1/*δ*. Such a fitting yields *δ* = 9.917(8) (see the inset to Fig. 5, a logarithmic plot of all MH data near *T*_C_ can be seen in Figure S2 in Supplementary Information). Using the Widom scaling relation 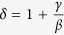 and the values of *β* and *γ* determined from Modified Arrott plot and Kouvel-Fisher plot, we obtain *δ* = 10.012(47) and *δ* = 9.925(56), respectively, which are very close to that obtained from critical isotherm analysis. Therefore, the critical exponents and *T*_*C*_ obtained in this work are self-consistent and accurate within the experimental precision.

**Figure 6:**
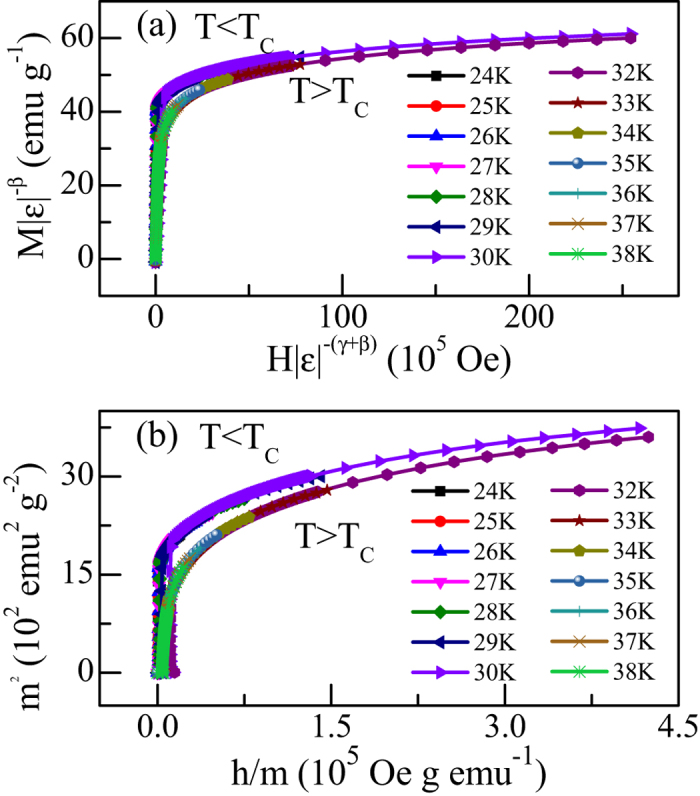
(**a**) The renormalized magnetization plotted as a function of renormalized field following Eq. (5) with *T*_*C*_ = 31.0 (K), and *β*, *γ* from Table 1 for CrSiTe_3_. (**b**) The renormalized magnetization and field (defined in text) replotted in the form of *m*^*2*^ vs. *h/m* for CrSiTe_3_. The above two plots show all data collapse into two separate branches: one below *T*_*C*_ and another above *T*_*C*_.

In order to further verify the values of the critical exponents and *T*_*C*_, we used Eq. (4) to check whether these critical exponents can generate a scaling equation of state for CrSiTe_3_. Figure 6(a) shows the plot of *m* vs. *h*. It can be clearly seen that all data collapse into two different curves: one below *T*_*C*_ and the other above *T*_*C*_. Additionally, we performed *m*^*2*^ vs. *h/m* plot in Fig. 6(b), where all data also fall on two independent branches. All these results clearly indicate that the interactions get properly renormalized in critical regime following scaling equation of state.

All critical exponents derived from various methods are summarized in Table 1 along with the theoretically predicted values for different models. The exponent *β* determined in this work is close to that reported in previous neutron scattering studies^14^,^15^. It is obvious that experimentally determined critical exponents *β*, *γ*, and *δ* are close to the 2D-Ising model. However, both *β* and *γ* show some deviation from the theoretical values, which might be associated with the following reasons. First, despite of strong 2D characteristics, CrSiTe_3_ has a 3D long-range ordering ground state owing to the non-negligible interlayer coupling^14^,^15^. Second, there is strong spin-lattice coupling in this material^13^. Both factors might contribute to the deviation from the prediction of the 2D Ising model.

Finally, we would like to discuss the nature as well as the range of interaction in CrSiTe_3_. For a homogeneous magnet, the universality class of the magnetic phase transition depends on the exchange interaction *J*(*r*). A renormalization group theory analysis predicts *J*(*r*) decays with distance *r* as^27^:


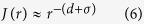


where *d* is the spatial dimensionality and *σ* is a positive constant. According to this model, the range of the spin interaction is long for *σ* < 2 and is short for *σ* > 2^27^. The susceptibility exponent *γ* is predicted as^27^:


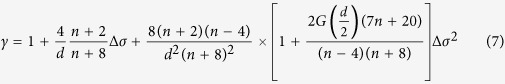


where 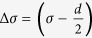 and 
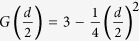
, *n* is the spin dimensionality. We followed the procedure similar to ref. 28 to get the range of interaction *σ* as well as the dimensionality of both lattice *d* and spin *n* in this system. The parameter *σ* is chosen for a particular values of {*d*:*n*} so that it yields a value of *γ* close to the experimentally observed *γ* = 1.562. The remaining exponents are then calculated from the following expressions: *ν* = *γ*/*σ*, *α* = 2 − *νd*, *β* = (2 − *α* − *γ*)/2, and *δ* = 1 + *γ*/*β*. This exercise is repeated for different set of {*d*:*n*}, and typical results are summarized in Table 2. It should be mentioned that the obtained exponents show significant difference from the experimentally determined critical exponents, when the spin is considered to be Heisenberg-like (*n* = 3), regardless of 2D (*d* = 2) or 3D (*d* = 3). This fact suggests that the spin interaction should not be of Heisenberg type. Nevertheless, {*d*:*n*} = {2:1} and *σ* = 1.630 give the exponents (*β* = 0.206, *γ* = 1.817, and *δ* = 9.811) which are close to our experimentally observed values (see Table 1). The value of *σ* = 1.630 suggests a long-range interaction with the attractive interactive interaction between the spins decaying with distance as *J*(*r*) ≈ *r*^−3.630^. Therefore, our results indicate that the spin interaction in CrSiTe_3_ is of 2D Ising type ({*d*:*n*} = {2:1}) coupled with long-range (*σ* = 1.630) interaction.

## Conclusion

In summary, we have reported a comprehensive study on the critical behavior of the PM-FM phase transition in the quasi-2D semiconducting ferromagnet CrSiTe_3_. The critical exponents (*β* = 0.170 ± 0.008, *γ* = 1.532 ± 0.001, and *δ* = 9.917 ± 0.008) and the critical temperature (*T*_C_ = 31.0 K) are determined using various techniques such as modified Arrott plot, Kouvel-Fisher plot, and the critical isotherm analysis. The consistency in the values of the critical exponents and the critical temperature obtained from different methods and the well-obeyed scaling behavior confirm that the obtained exponents are unambiguous and purely intrinsic to the material. The exponents determined in this study match well with those given by the renormalization group calculations for a 2D Ising system ({*d*:*n*} = {2:1}) coupled with long-range attractive interactions between spins decaying as *J*(*r*) ≈ *r*^−(*d*+*σ*)^ with *σ* = 1.630.

## Methods

Single-crystal samples of CrSiTe_3_ were prepared by the self-flux technique^13^. The structure and phase purity were confirmed by single-crystal and powder X-ray diffraction measurements at room temperature. The magnetization was measured using a Quantum Design SQUID-VSM magnetometer with the magnetic field applied parallel to the *c* axis of the sample. Isotherms were collected at an interval of 0.5 K around *T*_*C*_. Care has been taken to ensure that every curve was initially magnetized. The applied magnetic field *H*_*a*_ has been corrected by the demagnetization of the sample following the method described in ref. 29 and the corrected *H* was used for the analysis of critical behavior.

## Additional Information

**How to cite this article**: Liu, B. *et al*. Critical behavior of the quasi-two-dimensional semiconducting ferromagnet CrSiTe_3_. *Sci. Rep.* **6**, 33873; doi: 10.1038/srep33873 (2016).

## Supplementary Material

Supplementary Information

## Figures and Tables

**Table 1:** Comparison of critical exponents of CrSiTe_3_ with different theoretical models (MAP = Modified Arrott plot; KFP = Kouvel-Fisher plot; cal = Calculated).

Composition	Ref.	Technique	β	γ	δ
CrSiTe_3_	This work	MAP	0.170 ± 0.008	1.532 ± 0.001	10.012 ± 0.047^cal^
		KFP	0.175 ± 0.009	1.562 ± 0.009	9.925 ± 0.056^cal^
		Critical isotherm			9.917 ± 0.008
	14	Neutron	0.17		
	15	Neutron	0.151 ± 0.002		
2D Ising	20	Theory	0.125	1.75	15
Mean field	18	Theory	0.5	1.0	3.0
3D Heisenberg	18	Theory	0.365	1.386	4.8
3D XY	18	Theory	0.345	1.316	4.81
3D Ising	18	Theory	0.325	1.24	4.82
Tricritical mean field	19	Theory	0.25	1.0	5

The errors represent the fitting error.

**Table 2:** Critical exponents calculated following the renormalization group theory (see text).

d	n	σ	β	γ	δ
2	1	1.63	0.206	1.817	9.811
2	3	1.17	0.357	1.562	5.375
3	3	2.08	0.347	1.562	5.501
