# Supplementary material for: Critical behavior of the quasi-two-dimensional semiconducting ferromagnet CrSiTe3
Source: Sci Rep. 2016 Sep 21;6:33873. doi: 10.1038/srep33873 (PMC5030636; doi:10.1038/srep33873)
Supplement: Supplementary Information [file srep33873-s1.pdf]

Supplementary Information

Critical behavior of the quasi-two-dimensional semiconducting ferromagnet $CrSiTe_3$

Bingjie Liu,[1,2] Youming Zou,[1] Lei Zhang,[1] Shiming Zhou,[3] Zhe Wang,[1,2] Weike Wang,[1] Zhe Qu,[1,*] and Yuheng Zhang[1,3]

1. High Magnetic Field Laboratory, Chinese Academy of Sciences, Hefei, Anhui 230031, China.
2. University of Science and Technology of China, Hefei, Anhui 230026, China.
3. Hefei National Laboratory for Physical Sciences at Microscale, University of Science and Technology of China, Hefei, Anhui 230026, China.

Correspondence and requests for materials should be addressed to Z. Q. (email: zhequ@hmfl.ac.cn)

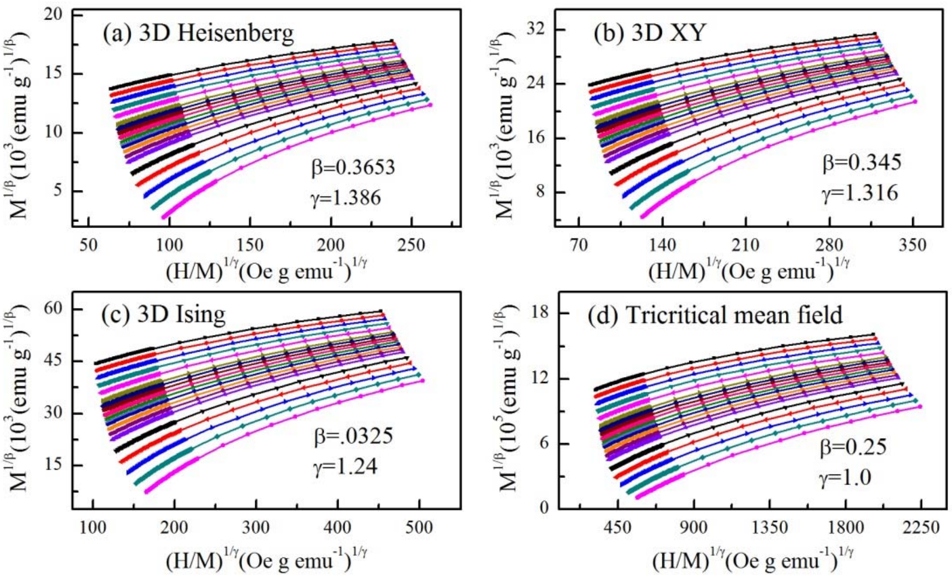

Figure S1. The isotherms replotted as $M^{1/\beta}$ vs. $(H/M)^{1/\gamma}$ with (a) 3D-Heisenberg model; (b) 3D-XY model; (c) 3D-Ising model; (d) tricritical mean-field model.

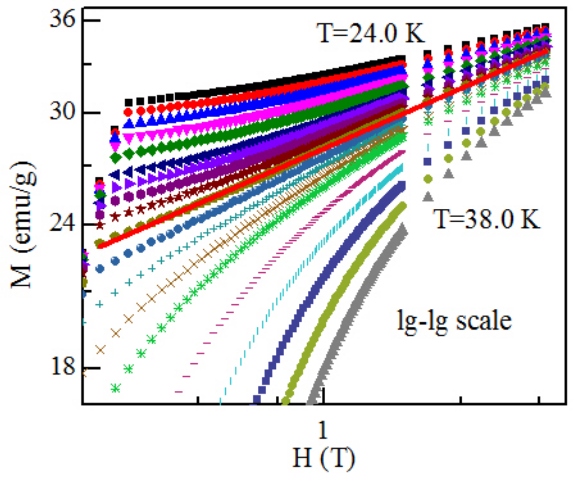

Figure S2. The isotherms replotted in lg-lg scale. The red solid line represents the fitting result to MH data at 31.0 K.

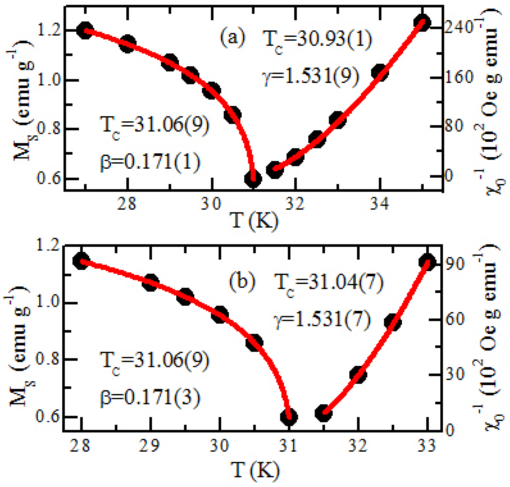

Figure 2. Temperature variation in spontaneous magnetization $M_S(T)$ (left axis) and inverse initial susceptibility $\chi_0^{-1}(T)$ (right axis) which are obtained from the high-field extrapolation of modified Arrott plot. The red solid lines represent the fitting to Eqs. 1 and 2 with **(a)** $\Delta T/T_C = 0.15$ and **(b)** $\Delta T/T_C = 0.10$, respectively.

$\Delta T/T_C$		β	γ	T_C (K)
0.20	$T < T_C$	0.170±0.008		31.06±0.09
	$T > T_C$		1.532±0.001	30.83±0.09
0.15	$T < T_C$	0.171±0.001		31.06±0.09
	$T > T_C$		1.531±0.009	30.93±0.01
0.10	$T < T_C$	0.171±0.003		31.06±0.09
	$T > T_C$		1.531±0.007	31.04±0.07

Table I. The critical exponents and the critical temperature of $CrSiTe_3$ determined by MAP technique with different $\Delta T/T_C$ values.
